# Investigation of the relationship between endothelial nitric oxide synthase T786C polymorphism and PSA, PSA derivatives, and prostate cancer in the Turkish population

**DOI:** 10.5937/jomb0-33122

**Published:** 2023-08-25

**Authors:** Senay Balci, Serin Akbayir, Murat Bozlu, Lulufer Tamer

**Affiliations:** 1 Mersin University, Faculty of Medicine, Department of Biochemistry, Yenisehir/Mersın, Turkey; 2 Kiziltepe State Hospital, Central Laboratory, Mardin, Turkey; 3 Mersin University Hospital, Department of Urology, Yenisehir/Mersın, Turkey

**Keywords:** endothelial nitric oxide synthase, prostate cancer, prostate-specific antigen, prostate-specific antigen derivatives, T786C polymorphism, endotelna sintaza azot oksid, rak prostate, antigen specifičan za prostatu, derivati antigena specifični za prostatu, polimorfizam T786C

## Abstract

**Background:**

Prostate cancer is a slowly progressing cancer. However, it has remained a major medical problem for affected men. Risk factors of prostate cancer include age, race, and prostate cancer family history. Prostate cancer may occur at different frequencies between ethnic populations and countries. Currently, studies on genetic risk factors in prostate cancer aetiology have been increasing. Due to the importance of changes in endothelial nitric oxide synthase in carcinogenesis, we aimed to reveal whether eNOS T786C polymorphism is associated with prostate cancer.

**Methods:**

Archival samples included in this study were whole blood samples taken from patients who were grouped according to prostate biopsy pathology results (BPH, n: 42; PCa, n: 48) and from healthy participants (controls, n:27). DNA was isolated from these whole blood samples and real-time polymerase chain reaction analysis was performed for endothelial nitric oxide synthase T786C polymorphism with LightCycler 480 II. Measured free and total prostate-specific antigen serum levels were evaluated retrospectively.

**Results:**

There was a statistical difference between patient-healthy control and control-healthy control groups regarding genotype distributions for eNOS T786C hism. Controls were more likely to have TC and CC genotypes and C alleles than the other two groups.

**Conclusions:**

Compared to other groups, the percentage of the eNOS786C allele in the control group was found to be higher. As a result of these data, it can be thought that carrying the allele may be protective against the disease.

## Abstract (sr)

**Uvod:**

Rak prostate je vrsta raka koji polako napreduje. Međutim, to je i dalje veliki medicinski problem za muškarce koji od njega boluju. Faktori rizika za rak prostate uključuju starost, rasu i porodičnu istoriju raka prostate. Rak prostate se može javiti sa različitom učestalošću među etničkim populacijama i zemljama. Trenutno je sve veći broj studija o genetskim faktorima rizika u etiologiji raka prostate. Zbog značaja promena u endotelnoj azot-oksid sintazi u kancerogenezi, želeli smo da otkrijemo da li je polimorfizam eNOS T786C povezan sa rakom prostate.

**Metode:**

Arhivski uzorci uključeni u ovu studiju su bili uzorci pune krvi uzeti od pacijenata koji su grupisani prema rezultatima patologije biopsije prostate (BPH, 42; PCa, 48) i od zdravih učesnika (kontrolni pacijenti, 27). Iz ovih uzoraka cele krvi izolovana je DNK i izvršena je analiza lančane reakcije polimeraze u realnom vremenu za polimorfizam endotelne azot-oksid sintaze T786C pomoću LightCicler 480 II. Izmereni nivoi slobodnog i ukupnog serumskog antigena specifičnog za prostatu su procenjeni retrospektivno.

**Rezultati:**

Postojala je statistička razlika u odnosu između pacijenata i zdravih kontrolnih subjekata i grupe kontrolnih u odnosu na zdrave kontrolne subjekte u pogledu distribucije genotipa za polimorfizam eNOS T786C. Kontrolni subjekti su češće imali TC i CC genotipove i C alele nego druge dve grupe.

**Zaključak:**

U poređenju sa drugim grupama, utvrđeno je da je procenat alela eNOS786C u kontrolnoj grupi veći. Kao rezultat ovih podataka, može se smatrati da aleli mogu da zaštite od bolesti.

## Introduction

Prostate Cancer (PCa) is the most common organ cancer in males worldwide [1]. It is one of the slow-progressing and silent cancers seen in advanced ages and started to be diagnosed at an earlier age due to increasing screening studies in recent years. Despite progress, PCa remains a major medical problem for affected men, so research into the risk factors, early diagnosis and treatment of the disease is ongoing [2] [3]. Well-known risk factors for PCa are a history of PCa in the family, age, and race. Although its aetiology is not clear, it is known to occur at different frequencies between ethnic populations and countries [2] [4]. Additionally, differences in PCa cases and outcomes have been observed among men of different racial groups. PCa incidence and poor prognosis are higher in some races [3] [5]. Recently, the genetic risk factors studied and found in PCa have raised interest in the search for common genetic variants.

For the early diagnosis of PCa, serum prostatespecific antigen (PSA) level and digital rectal examination (DRE) combination are recommended as annual screening after the age of 50 [6] [7] [8]. In the presence of a suspicious mass in the prostate, transrectal ultrasound (TRUS) guided biopsy is the gold standard method for diagnosing PCa [8]. However, since there are cases in which PSA is insufficient, and prostate biopsy is an interventional procedure, research continues for new diagnostic biomarkers.

Two of the molecules investigated are Nitric Oxide (NO), which has been shown to be related to the aetiology of PCa, and nitric oxide synthase (NOS), the enzyme that enables it to be synthesised [1] [4] [9]. Some studies have shown that NO levels rise with inflammation and malignancy and may have a bimodal behaviour in PCa development [10]. It has been reported that endothelial NOS (eNOS, NOS3), one of its NOS isoenzymes, is vital in vascular improvement and carcinogenesis. It has been suggested that varied genetic polymorphisms in the eNOS gene already defined may be responsible for variations in the genetic control of NO levels [4] [10] [11].

Since the exact aetiology of PCa is not entirely understood, research on PCa continues. PCa usually has a poor prognosis, and it is thought that the links between tumour development and clinical outcomes can be determined by genetic diversity to determine prognosis [9] [10]. Detecting the presence of genetic changes can be a useful tool as a molecular indicator of PCa prognosis. In light of this information, this study aims to evaluate the status of eNOS T786C polymorphism in males with PCa and prostate benign diseases and reveal its possible relationship with PCa. This study also aims to determine whether eNOS T786C polymorphism is a risk factor for PCa. In addition, if there is a relationship between eNOS T786C polymorphism with PSA and its derivatives, we also plan to show it.

## Materials and methods

This study was approved by the Mersin University Clinical Research Ethics Committee dated November 25, 2020, numbered 2020/758.

### Working group and sampling

One hundred seventeen patients’ samples were included in the study; 48 samples were grouped as the patient group, 42 as the BPH group, and 27 as the healthy control group. Archive samples included in this study were whole blood samples taken from patients who applied to the urology outpatient clinic, had a prostate biopsy and were grouped according to pathology results. According to prostate pathology results, patients diagnosed with the malignant prostate disease were included in the patient group (mean age: 62.83±7.96), and those diagnosed with benign prostate diseases were included in the BPH group (mean age: 65.58±8.99; PSA: 2–10 µg/L). The patient group were newly diagnosed with PCa, did not take medication for PCa, were not hospitalised for another malignancy or chronic disease in the past year, and did not have distant metastases. The BPH group did not take medication for other diseases and were not hospitalised for another malignancy or chronic disease in the past year. In the healthy control group participants (mean age: 64.63±11.25), the prostate biopsy was not performed, and a PSA value between 2–10 µg/L and with no prostate cancer family history, no drug use, no chronic disease, and no hospitalisation history.

Free PSA (fPSA) and total PSA (T-PSA) serum levels were measured in Advia Centaur XP (Siemens Healthcare Diagnostics Inc, Tarrytown, NY, 10591- 5097, USA) hormone autoanalyser using the direct chemiluminometric sandwich immunoassay method, f/T PSA ratio, and fPSA% values were analysed and evaluated retrospectively. The reference range of T-PSA was 0–4 µg/L.

### DNA isolation and eNOS T786C polymorphism genotyping by RT-PCR

DNA isolation from archive whole blood taken in ethylene diamine tetraacetic acid (EDTA) containing tubes was performed in accordance with the manufacturer’s instructions (High Pure PCR Template Preparation Kit, Roche, Germany) and stored at 4°C.

Primers used for eNOS T786C polymorphism analysis were synthesised by standard phosphoramide chemistry (Ella Biotech GmbH, Planegg, Germany), and all fluorophore-labelled probes were synthesised by Metabion and purified by reverse-phase HPLC:

- Forward Primer: 5’-CCACCAGGGCATCA-AGCT- 3’
- Reverse Primer: 5’-CGCAGGTCAGCAGAGAG-ACTA- 3’

The Vic probe and fam probe were used to detect the T786C mutation. 5 ends of the Vic probe were marked with Yakima Yellow. Fam probe was a 13-mer oligonucleotide:

- 5’-Yakima Yellow-CTGGCTGGCTGAC-3’
- 5’-Fam-TCCCTGGCCGGCT-3'

PCR conditions for eNOS T786C polymorphism analysis were as follows:

- Denaturation for 600 seconds at 95°C;
- Denature 15 seconds at 95°C;
- Annealing for 60 seconds at 60°C (40 cycles);
- Extension for 30 seconds at 40°C.

eNOS T786C polymorphism was analysed with allelic discrimination with RT-PCR (LightCycler 480 II, Roche Diagnostics GmbH Mannheim, Germany).

### Statistical analysis

Analysis of data was carried out using a trial version of IBM SPSS Statistics Processor (IBM SPSS Software, Armonk, New York, United States).

Comparisons of more than 2 groups in terms of age variable were evaluated with ANOVA.

Control of the normal distribution of numerical PSA variables obtained from these individuals was performed by Kolmogorov-Smirnov and Shapiro-Wilk normality tests. PSA variables that were not compatible with normal distribution were summarised with median percentages (25%–75%) and minimum-maximum statistics. In comparing the two groups in terms of PSA and its derivatives, the non-parametric two independent groups Mann-Whitney U test was used. Comparisons of more than 2 groups in terms of variables were evaluated with Kruskal-Wallis statistics. Pearson Chi-square test results were used to control the relationship between categorical variables. Hardy- Weinberg (HW) balance test was used to examine the distribution of alleles by genotypes and the compatibility of this distribution with expected values. Possible risks of genotypes and alleles were determined by calculating the ODDS ratio. The statistical significance level was determined as p<0.05.

## Results

Age values in all groups showed a homogeneous distribution. The ratio of Hypertension, Diabetes Mellitus, other diseases, and PCa presence in the family in the groups are given in Table 1.

**Table 1:** The ratio of Hypertension, Diabetes Mellitus, Other Diseases, and Prostate Cancer presence in the family in the groups. HT: Hypertension, DM: Diabetes Mellitus, PCa: Prostate Cancer.

Group		HT %	DM %	Other <br>Diseases	PCa in <br>Family %
Patient	+	37.5	12.5	43.8	17.8
	-	62.5	87.5	56.2	82.2
BPH	+	33.9	15.3	44.8	32.1
	-	66.1	84.7	55.2	67.9
Healthy <br>Control	+	0	0	0	0
	-	100	100	100	100

There was a significant difference between the control and patient groups in terms of T-PSA and fPSA variables (p<0.001). There was no significant difference between the groups in terms of f/T PSA and fPSA% variables (p=0.277). A significant difference was found between the patient and BPH groups in terms of T-PSA, f/T PSA and fPSA% variables (p<0.001). However, there was no significant difference between the groups in terms of fPSA variable (p=0.353) (Table 2). When we analysed PSA and its derivatives’ levels, a significant difference was found between the healthy control and BPH groups in terms of T-PSA and fPSA variables (p<0.001). As in comparing the patient–healthy control groups, there was no significant difference between the groups in terms of f/T PSA and fPSA% variables (p=0.219). In all three groups, in terms of PSA and its derivatives between eNOS T786C polymorphisms, no statistical difference was observed (p>0.05) (Table 2).

**Table 2:** Summarising and analysis of PSA and its derivatives measurement values in patient and healthy control groups. ^a^ patient-control comparison p=<0.001 <br>^b^ patient-BPH comparison p=<0.001 <br>^c^ control-BPH comparison p=<0.001 <br>T-PSA: total prostate specific antigen, fPSA: free prostate specific antigen, f/T PSA: free/total PSA ratio, fPSA %: free PSA percentage. <br>The results were reported as mg of PSA per liter (mg/L) of blood.

Group		T-PSA	fPSA	f/T PSA	fPSA %
Control	Minimum	0.23	0.07	0.07	7.06
	Maximum	4.34	0.95	0.42	41.80
	% 25	1.87	0.29	0.13	12.86
	% 50	2.33^a,c^	0.44^a,c^	0.20	20.12
	% 75	3.26	0.71	0.30	30.29
BPH	Minimum	2.39	0.01	0.002	0.18
	Maximum	8.91	2.87	0.48	47.70
	% 25	4.84	0.91	0.19	18.51
	% 50	6.01^b,c^	1.41^c^	0.23^b^	22.59^b^
	% 75	7.27	1.83	0.30	29.53
Patient	Minimum	3.49	0.48	0.04	4.36
	Maximum	100.00	24.28	0.41	41.1
	% 25	5.56	0.92	0.12	11.99
	% 50	9.14^a,b^	1.49^a^	0.17^b^	16.75^b^
	% 75	18.05	2.32	0.23	22.75

The ratios of eNOS T786C polymorphism genotypes in groups are given in Table 3. The percentage of TC genotype (p=0.006) and CC genotype (p=0.023) was found to be higher in the control group compared to the patient group. There was also a significant difference in allelic distributions for the eNOS T786C polymorphism (p=0.016). The probability of having the C allele in healthy controls was 2.37 times higher than in the patient group (p=0.017). There was no statistical difference between the patient and BPH groups regarding genotype distributions for eNOS T786C polymorphism (p=0.646). There was no statistical difference between the patient and BPH groups in terms of allele distributions for eNOS T786C polymorphism (p=0.264). The patient group was 1.40 times more likely to have a C allele than the BPH group. Again, this rate was not statistically significant (p=0.264). A statistically significant difference was found between the healthy control and BPH groups regarding genotype and allele distributions for the eNOS T786C polymorphism (p=0.001). Compared to the BPH group, the control group was more likely to have the TC genotype (p=0.004) and CC genotype (p=0.008) and was 3.32 times more likely to have the C allele (p=0.001) (Table 3).

**Table 3:** Chi-square and ODDS Ratio results concerning eNOS T786C polymorphism genotypes and alleles in the groups. a patient-control comparison <br>b patient-BPH comparison <br>c control-BPH comparison <br>n: number, eNOS: Endothelial Nitric Oxide Synthase, OR: ODDS Ratio.

eNOS T786C	Control <br>(n=27)		BPH <br>(n=42)		Patient <br>(n=48)		X^2^	OR	
	n	%	n	%	n	%	p	OR <br>(95% CI)	p
TT (Wild)	1	3.7	19	45.2	18	37.5	0.003^a^ <br>0.646^b^ <br>0.001^c^	1.00	——
TC	14	51.9	11	26.2	12	25.0		21.00 (2.43–181.42)^a^<br>1.15 (0.41–3.26)^b^ <br>24.18 (2.79–209.77)^c^	0.006^a^ <br>0.791^b^ <br>0.004^c^
CC	12	44.4	12	28.6	18	37.5		12.00 (1.41–102.21)^a^<br>1.58 (0.60–4.19)^b^ <br>19.00 (2.18–165.46)^c^	0.023^a^ <br>0.355^b^ <br>0.008^c^
Allele Frequency									
T	16	29.6	49	58.3	48	50.0	0.016^a^ <br>0.264^b^ <br>0.001^c^	——	
C	38	70.4	35	41.7	48	50.0		2.37 (1.17–4.82)^a^ <br>1.40 (0.78–2.53)^b^ <br>3.32 (1.61–6.88)^c^	0.017^a^ <br>0.264^b^ <br>0.001^c^

## Discussion

According to this study’s results, the ratios of eNOS T786C polymorphism genotypes (total of TC and CC) were close to each other in the patient and BPH groups, but the healthy control group’s ratio was higher than the other groups. As a result of the within- group comparison in all three groups, no significant difference was detected between eNOS T786C polymorphisms genotype distribution and PSA and its derivatives. There was a statistically significant difference between patient-control and BPH-control groups regarding genotype distributions for eNOS T786C polymorphism. Controls were more likely to have TC, CC genotypes, and C alleles than the other two groups. In the third comparison, the patient group was more likely to have TC and CC genotypes and C alleles than the BPH group, but there was no significant difference.

Various studies have shown that eNOS is effective in cancer-related processes such as angiogenesis, apoptosis, invasion and metastasis. It has been suggested that the eNOS T786C polymorphism affects the carcinogenesis process by causing mutant alleles, altered eNOS activity and NO concentrations. Although there are studies investigating the relationship between eNOS polymorphisms and cancer risk, the results appear to be conflicting [4] [12].

Abedinzadeh et al. [9] showed that the eNOS T786C polymorphism was significantly associated with increased PCa risk in the general population, especially in Caucasians, as a result of a meta-analysis including five studies. In the study of Polat et al. [1], it was suggested that the TC genotype was important for the eNOS T786C polymorphism in Turkish patients with PCa. Again, in the study of Diler and Öden [13] conducted in the Turkish population, they showed that the genotype and allele frequencies of the eNOS T786C polymorphism in PCa patients were statistically significant, and their findings suggested that the eNOS T786C polymorphism might be associated with PCa sensitivity in the Turkish population. Another study found that the CC genotype for the eNOS T786C polymorphism increased the risk of PCa and was associated with an increased rate of high-grade and advanced disease [14]. Similar to Safarinejad’s et al. [14] findings, in the meta-analysis of Zhang et al. [15], it was concluded that the CC genotype of the eNOS T786C polymorphism was associated with cancer risk, especially in the Caucasian population. Some studies showed that individuals with the C allele for the eNOS T786C polymorphism reduced the promoter activity of the eNOS gene responsible for endothelial NO production compared to the T allele [16]. Although TC and CC genotypes were observed more frequently in PCa patients compared to benign prostate diseases, we found that TC and CC genotypes were more common in the healthy control group compared to the other groups, contrary to previous data.

In a meta-analysis, ORs from 11 studies associated with the eNOS T786C polymorphism were combined. In the subgroup analysis based on ethnicity, high cancer risk was found in Caucasians but not in Asians, and this difference was associated with a small number of studies conducted on Asians with different ethnic backgrounds. When stratified by cancer type, a significant relationship was found between the eNOS T786C polymorphism and the increased risk of PCa [17].

It has been suggested that a dose-dependent relationship exists between NOS expression and cancer response. It has been reported that high concentrations of NO have an anti-neoblastic effect by causing apoptosis, and in low concentrations, proangio genic and pro-cancerous effects predominate by protecting cells from apoptosis. Some studies have shown that endogenous NO levels were higher in cancerous prostate tissue than in normal tissue. Conversely, in another study, NOS expression decreased in BPH developing in the transitional zone of the prostate, and consequently, there was a decrease in NO production [18] [19] [20].

Since the eNOS T786C polymorphism is located in the gene’s promoter region, it is important to bind RNA polymerase to this region and the gene’s transcription. Polymorphism in this region was thought to trigger the development of PCa by causing the increase or decrease of NO synthesis, although the underlying mechanisms are not fully known [1] [9]. It has been suggested that NO may be associated with tumour development as a result of stimulation of angiogenesis and increased mutagenesis by direct activation of free radicals on DNA. In addition, it has been said that NO release from tumour cells may result from the role of NO in tumour-induced immunosuppression through its anti-proliferative effect. According to the results obtained from these, it has been said that the increase in NO may be a result of PCa and a cause of PCa [21] [22]. In a study comparing the level of NO in the serum of BPH and PCa patients, it was observed that NO levels were increased in patients with BPH and PCa, but it was shown that the increase was higher in patients with PCa. In addition, it was thought that the significant decrease in NO levels in the radical prostatectomy group might be due to a correlation between PCa and serum NO levels [23]. Considering the results of all these studies, contradictory results are seen in NO levels, such as eNOS expression.

When we evaluated the results of this study, we could not find a relationship between eNOS T786C polymorphism genotypes and PSA, PSA derivatives, and PCa. However, the percentage of the C allele in the control group was found to be higher in genotype and allele comparisons. These data show that the risk of prostate cancer is low in those carrying the C allele, suggesting that carrying the C allele may be protective against the disease. Considering that no tumour marker can clearly distinguish between benign and malignant, the high rate of the C allele compared to the control group is a significant result.

Although several case-control studies have been conducted to assess the role of eNOS gene polymorphisms in susceptibility to PCa in different populations, conflicting results have been reported due to the relatively small sample size of the individual studies and the effects of the sample group. Apart from this, racial differences in polymorphism studies significantly affect the results.

The selected group may have caused the data differences in the literature, the number of participants, the use of different samples, and the difference in the participants selected as controls (such as choosing among participants with benign prostate disease such as BPH, chronic or acute inflammation, or healthy participants), the average age of the participants. We believe that the inconsistencies in the literature can be overcome by studies involving more participants, perhaps different racial subgroups, subdividing when necessary, and simultaneously measuring NO levels.

## Dodatak

### List of abbreviations

PCa, prostate cancer;<br>PSA, prostate-specific antigen;<br>DRE, digital rectal examination;<br>TRUS, transrectal ultrasound; <br>NO, nitric oxide;<br>NOS, nitric oxide synthase;<br>nNOS, NOS1, neuronal NOS;<br>iNOS, NOS2, inducible NOS;<br>eNOS, NOS3, endothelial NOS;<br>BPH, benign prostatic hyperplasia;<br>fPSA, free PSA;<br>TPSA, total PSA;<br>fPSA%, percentage of free PSA;<br>f/T PSA, free/total PSA ratio;<br>EDTA, ethylene diamine tetraacetic acid;<br>HW, Hardy- Weinberg Balance Test;<br>HT, hypertension;<br>DM, diabetes mellitus.

### Acknowledgement

We thank H. Didem OVLA for the statistical analysis of our research.

### Statement of Ethics

This study was approved by the Mersin University Clinical Research Ethics Committee dated November 25, 2020, numbered 2020/758.

### Author Contributions

SB planned the work, performed the analysis, evaluated the results, and wrote this article; SA planned the work, made a selection of samples, evaluated the results, and helped to write this manuscript; LT participated in its design and coordination, and helped to draft the manuscript; MB made a selection of samples, helped to evaluate the results of the analysis, and helped to draft the manuscript. All authors read and approved the final manuscript.

### Funding Sources

All expenses of the study were covered by the researchers.

### Conflict of interest statement

All the authors declare that they have no conflict of interest in this work.
